# Copaiba oil-resin (*Copaifera reticulata Ducke*) modulates the inflammation in a model of injury to rats’ tongues

**DOI:** 10.1186/s12906-017-1820-2

**Published:** 2017-06-14

**Authors:** Francisco Bruno Teixeira, Raíra de Brito Silva, Osmar Alves Lameira, Liana Preto Webber, Roberta Souza D’Almeida Couto, Manoela Domingues Martins, Rafael Rodrigues Lima

**Affiliations:** 1 Laboratory of Functional and Structural Biology, Institute of Biological Science, Federal University of Pará, Belém, 66075-900 Brazil; 2 Laboratory of Biotechnology, EMBRAPA, Amazonia Oriental, Belém, PA Brazil; 3 Department of Oral Pathology, School of Dentistry, Laboratory of Bucal Pathology, Dentistry College, Federal University of Rio Grande do Sul, Porto Alegre, Rio Grande do Sul Brazil

**Keywords:** Copaiba oil-resin, Inflammation, Complementary therapies, Macrophage, Model of injury, Amazonian biodiversity, *Copaifera Reticulata Ducke*

## Abstract

**Background:**

The regeneration of integrity and tissue homeostasis after injury is a fundamental property and involves complex biological processes fully dynamic and interconnected. Although there are medications prescribed to accelerate the process of wound healing by reducing the exaggerated inflammatory response, comes the need to search for different compounds of Amazonian biodiversity that can contribute to the acceleration of the healing process. Among these products, the copaiba oil-resin is one of the most prominent feature in this scenario, as they have been reported its medicinal properties.

**Methods:**

Aiming to evaluate the anti-inflammatory and healing effect of copaiba oil-resin (*Copaifera reticulata Ducke*) in transfixing injury of rats’ tongues first proceeded up the copaiba oil-resin oral toxicity test in 5 male mice to stipulate the therapeutic dose which was established at 200 mg/kg/day. Then it was induced transfixing injury in a total of 15 Wistar rats. The animals were randomly divided into three groups based on the treatment: control group, dexamethasone group and copaiba oil-resin group. After 7 days of treatment, histological slides stained with hematoxylin and eosin was prepared. Immunohistochemistry for CD68 (macrophage marker) was performed and analyzed by the cell counter Image J.

**Results:**

The acute toxicity test showed that the oil-resin copal has low toxicity. Furthermore, copaiba oil-resin therapy modulates the inflammatory response by decreasing the chronic inflammatory infiltrate, edema and specifically the number of macrophages.

**Conclusions:**

The results indicate the potential of the Amazon region and showed up relevant because therapy with this extract modulates the inflammatory process.

## Background

The Brazilian Amazon concentrates a large amount of natural resources with various therapeutic applications in alternative medicine. The oil-resin, copaiba, is a natural product of the Amazon’s biodiversity more highlighted in this scenery [1, 2], mainly due to its anti-inflammatory and healing properties [2, 3]. Copaiba oil-resin is an extracted exudate from trees in the genus *Copaifera* (“copaibeiras”) that has more than 60 species cataloged in the world [3]. Copaiba oil-resin is one of the most important renewable natural remedy for the populations of the Amazon region [2].

Studies with copaiba oil-resin showed that this natural product is formed by several components of the bases diterpenes and sesquiterpenes, and the concentrations of these compounds can vary according to species, soil properties, climatic conditions, and the period of the year when the extraction was done [3].

Copaiba oil-resin is often used orally or topically [2]. Several studies using different species and in various administration forms identified its anti-inflammatory and wound healing property in different parts of the body [4–10].

In the Amazon region, the species, *Copaifera reticulata Ducke,* is the most frequently found and the most often used. Due to this, copaiba oil-resin is found in very popular street markets indicated treatments with injured due to their possible anti- inflammatory activity and healing [2].

Due to its widespread use, some scientific studies have proposed identify the main therapeutic activities that this compound had. From this, some studies in vitro and in vivo have observed its larvicide activity [11] and analgesic [12], antioxidant [2], anxiolytic [13], and antimicrobial [14], and inhibition of microglial and neutrophilic activation [15]. In addition to these properties, some studies have found the use of this species to be safe, and neither high toxicity nor teratogenic activity in pregnancy has been observed [16, 17].

Considering the complexity of repair tissue physiology and the structural and functional damage incurred due to wounds, it is necessary to search for different compounds that can contribute to the reduction of the inflammatory response and to accelerate the healing process. Our study evaluates the anti-inflammatory properties of copaiba oil-resin (*Copaifera reticulata Ducke*) in a model that transfixes injury in rats’ tongues.

## Methods

### Plant material, oil-resin extraction and caracterization

For oil-resin collection were used dispersed native adult plants of *Copaifera reticulata Ducke* over 30 years old located in the municipality of Belterra, Pará, Brazil. The trees were randomly drilled with a traditional auger 2 cm diameter and 45 cm in length, making up two holes in the height of 1 m and 1.50 m, respectively. Oil-resin samples were stored in plastic containers (2000 ml) by the action tree and protected from light with aluminum foil. Subsequently, they were transferred to glass jars (20 ml) for further analysis. The bore of the tree after the complete draining of the oil-resin pipe was sealed with PVC type with a diameter of ¾ and 10 cm long containing a plastic cap in order to facilitate collection and avoid other waste wood. A copy of the plant species deposited in the Herbarium IAN EMBRAPA Eastern Amazon (Exsicata: 183,939). The method of chemical characterization of copaiba oil-resin has been described by Santos-Guimarães et al., 2012 [15].

### Experimental animals

A total of 15 adult male Wistar rats (3 months old), obtained from the Federal University of Pará (UFPA) were kept in collective cages (5 animals per cage) and more 5 mice (*Mus musculus*) adult male *Swiss.* Animals were maintained in a climate-controlled room with a 12 h light-dark cycle (lights on 7:00 AM) and food and water ad libitum. All procedures were approved by the Ethics Committee on Experimental Animals of the Federal University of Pará (license number BIO-203-14), following the *Guide for the Care and Use of Laboratory Animals*.

### Acute oral toxicity test (OECD, 2008)

A test for acute oral toxicity was performed, according to the Guide 425/2008 from the Organization for Economic Co-operation and Development (OECD) [18], where the estimation of the LD50 was estimated using the limit test method. In this study, a single Swiss albino mouse (*Mus musculus*), weighing 20 to 30 g, received a dose of 2000 mg/kg and were observed for 48 h. If the animal survived, the other 4 animals were sequentially tested with the same dose. The survival of 3 or more animals qualifies as the LD50 greater than 2000 mg/kg (low toxicity).

If the first animal should die or deaths were greater than or equal to three of five animals, would follow-up for the main test. Another animal received 175 mg/kg orally. If the animal survived, another animal was tested with a higher dose (range of 3.2×) until a dose resulted in death or overt toxicity. When the first animal of the main test manifested obvious intoxication or death, the following animal received a lower dose (range of 3.2×). The test continued until 3 consecutive animals survived the upper limit dose (2000 mg/kg) or 5 recoveries occurred in any of the 5 animals tested consecutively, and at least 4 animals recovered.

In this model, all animals were observed individually for 30 min after administering the dose and then evaluated periodically during the 48 h following surgery. In this evaluation, toxic manifestations were observed, such as changes in skin, fur, eyes, and mucous membranes. Toxicity was also evident in the animals’ circulatory, respiratory, autonomic and central nervous systems and in locomotor behavior. The tests evaluating copaiba oil-resin effects were carried out with doses equivalent to 10% of the lowest dose that caused the deaths of at least three animals.

### Induction of transfixing injury and experimental groups

The transfixing lesions were induced by first placing an Ainsworth clamp on the previous third of the tongue. The animals were anesthetized with ketamine (90 mg/kg, ip) and xylazine (10 mg/kg, ip). Soon after, the animals were positioned supine on a flat area and had the tongue exposed. With the aid of two Perry tweezers, which bored into the body to produce injury, two perforations were carried out: one in the right lobe of the tongue and one on the left lobe to form standard, traumatic injuries. After surgery the animals were kept with water and food ad libitum in individual cages and divided into the following groups: a control group, a dexamethasone group, and a copaiba oil-resin group, each group *n* = 5. All animals were let to recover for 7 days.

### Animal treatment

Twelve hours after surgery, animals assigned to the copaiba oil-resin group received 10% of the dose set by the acute oral toxicity test; the control group received a dose of Tween® 20 corresponding to the volume of the dose given to the animals in the copaiba oil-resin group. Animals of the third group were treated with dexamethasone and received a dose of 0.5 mg/kg/day [19]. All treatments were performed by intragastric gavage (oral route).

### Obtaining of samples and histological procedures

**Fig. 1:**
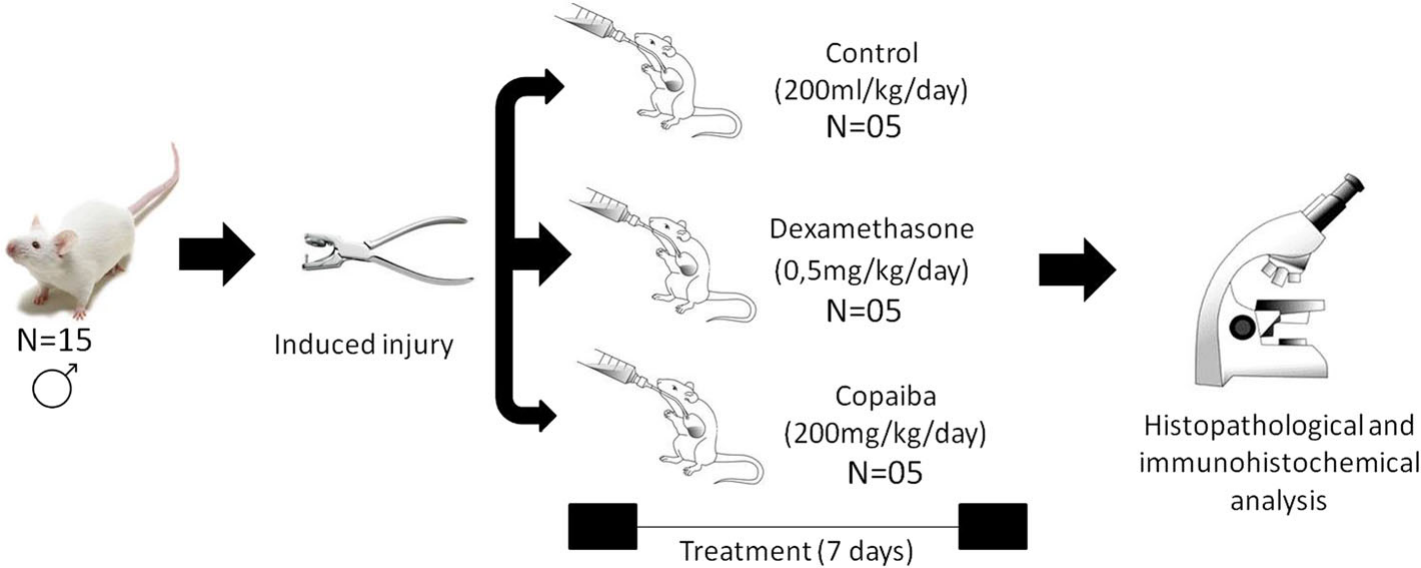
Schematic representation of the experimental design utilized in the present study

After the stipulated survival time, animals were deeply anesthetized with ketamine (90 mg/kg, ip) and xylazine (10 mg/kg, ip) and euthanized by cervical dislocation. Tongues were removed and fixed in 10% formalin for at least 48 h. After this period, the specimens were washed with distilled water, dehydrated in progressive concentrations alcohol solutions, cleared in xylene, and embedded in paraffin. Five μm sections were obtained for hematoxylin and eosin (HE). Figure 1 depicts the schematic drawing of the applied methodology.

### Histopathological evaluation

The qualitative and semi-quantitative analyses of HE stained slides were performed by two blinded examiners using a conventional light microscope. For qualitative analysis (descriptive evaluation) the following histopathological features were described: edema, inflammatory infiltrate, angiogenesis and muscle fibers. After that, semi-quantitative analysis (scores evaluation) of edema and inflammatory infiltrate were performed using a grading system: Grade 0 (absent), Grade 1 (mild), Grade 2 (moderate) and Grade 3 (severe or intense) based on Wagner. 2013 [20] and Coelho. 2015 [21]. The examiners were previosly calibrated and Kappa coefficient test (*p* > 0.7) were calculated to determine the concordance of score analysis.

#### Immunohistochemistry

For immunohistochemical staining, the samples were sectioned into 3-μm sections and placed on silanized slides. The slides were deparaffinized in xylene and hydrated in descending grades of ethanol. Antigen retrieval was performed prior to incubation of the primary antibody. The primary antibody, sources, antigen retrieval, dilutions and incubation times were as follows: CD68 (ED1, AbD Serotec, low pH solution in a water bath at 90 °C for 18 h, 1:100, 2 h). The sections were then incubated with diaminobenzidine tetrahydrochloride (DAB, Novocastra, Newcastle, UK) and counterstained with Mayer’s hematoxylin. Negative controls were obtained by replacing the primary antibody with non-immune serum. The positive control were sections of human tonsil for CD68.

All immunohistochemical analysis was performed by one blinded observer of the treatment received. Five images of the wound healing area were captured at a magnification of 400× using a conventional light microscope (CX41RF model, Olympus Latin America, Inc., Miami, Florida, USA) coupled to a camera (QColor 5, RTV, Olympus Inc., BX51, Canada) and connected to a computer (Dimension 5150, Dell, Porto Alegre, RS, Brazil) using the QCapture software program, version 2.81 (Quantitative Imaging Corporation, Inc., Surrey, British Columbia, Canada). The images were analyzed using Image J software program (National Institute of Mental Health, Bethesda, Maryland, USA). The slides, stained with CD68, were analyzed and only the cells with cytoplasmic brown labeling were considered positive.

### Statistical analysis

The results obtained in the various assessments were entered into GraphPad Prism 5.0 software. The nonparametric Kruskal-Wallis test was used with a significance level of *p* < 0.05 for evaluation of data for scores. For quantitative evaluation of immunohistochemical assays we used ANOVA with post 1 via a Tukey test. Results were expressed textually and graphically with means and standard error (mean ± SE).

## Results

### Oil-resin characterization

The copaiba oil-resin used in this research was extracted from the species, *Copaifera reticulata Ducke,* and has been characterized by Guimarães-Santos et al. in 2012 [8]. For this study, 27 components were identified with the following concentrations: δ-elemene (0.2%), cyclosativene (0.9), α-copaene (0.5%), δ-elemene (0.2%), cyclosativene (0.9%), α-copaene (0.5%), β-elemene (3.2%), α-gurjunene (0.7%), β-caryophyllene (37.3%), trans-α-bergamotene (9.0%), aromadendrene (0.9%), epi-β-santalene (0.1%), α-humulene + (E)-β-farnesene (5.4%), β-chamigrene (1.0%), γ-gurjunene (0.6%), γ-curcumene (0.6%), β-selinene (4.8%), α-selinene (3.0%), (Z)-α-bisabolene (1.8%), α-bulnesene (2.2%), β-bisabolene (14.5%), β-curcumene (0.4%), β-sesquiphelandrene (1.2%), (E)-γ-bisabolene (1.4%), caryophyllene oxide (0.1%), epi-β-bisabolol (0.1%), and β-bisabolol (0.2%).

### Acute oral toxicity test

On the acute oral toxicity test, none of the 5 animals tested with the limit dose (2000 mg/kg/day) died or had any sign or symptom of toxicity during the 48-h test. Thus, the dose prescribed for this study was 10% of the limit dose, i.e., the copaiba oil-resin group of animals received 200 mg/kg/day and the control animals received 200 mL/kg/day of Tween 20.

### Qualitative histopathological analysis

The proposed lesion induction model produced an extensive wound to the two surfaces of the tongue, reaching all the constituents of the animals’ body tissue, including epithelial tissue, connective tissue, and muscle tissue. The transfixing injury promoted a deep character injury.

Thus, the major differences were found in a histopathological analysis at this deepest portion of the wound, particularly in the muscle tissue of all groups.

**Fig. 2:**
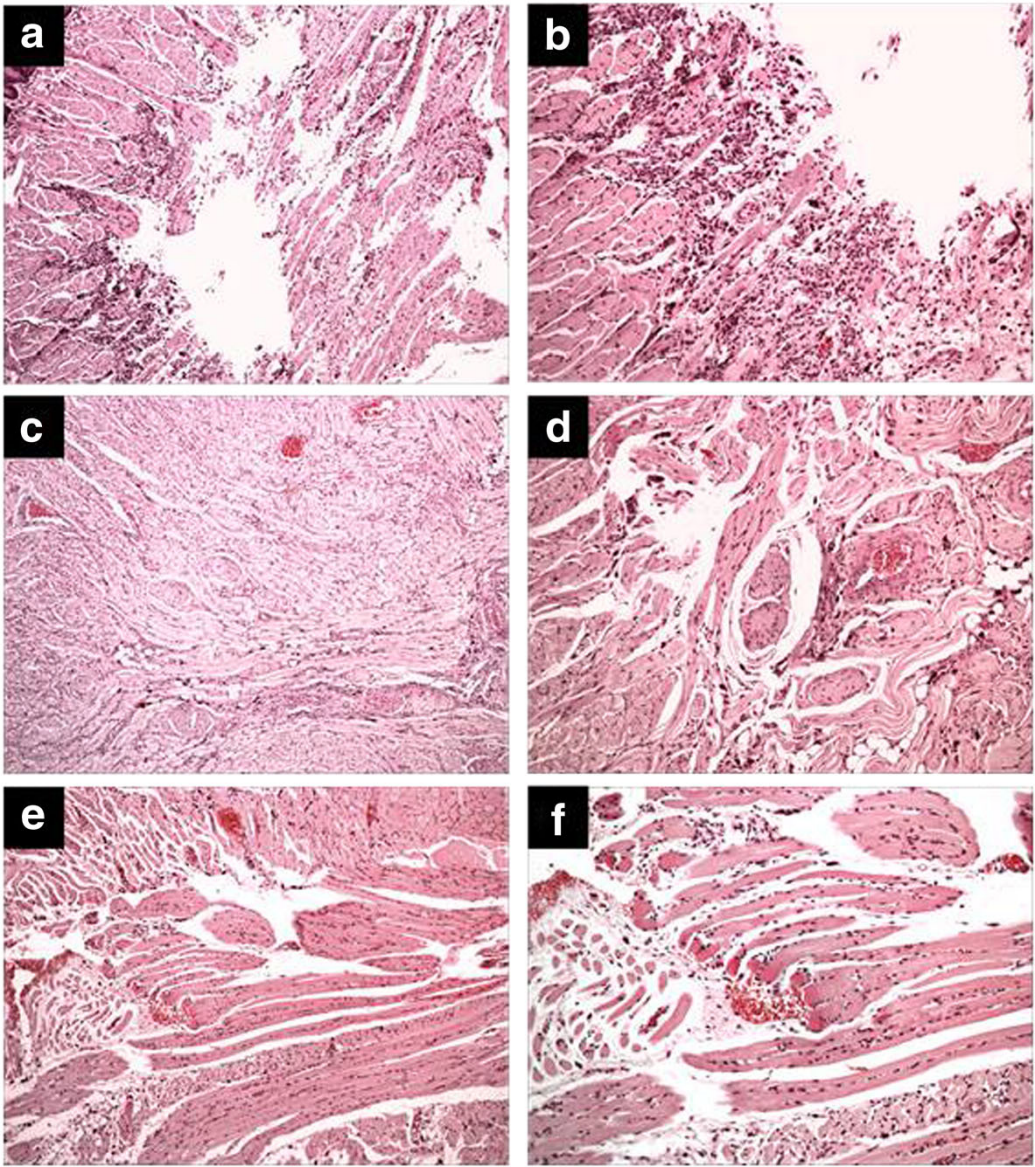
Effect of treatment with copaiba oil-resin on transfixing lesions in rats’ tongues stained by hematoxylin and eosin (HE). Panels (**a** and **b**) represent the control group. Panels (**c** and **d**) represent the dexamethasone group. Panels (**e** and **f**) represent the copaiba oil-resin group. It is found that the groups dexamethasone and copaiba oil-resin showed similar aspects decreasing chronic inflammatory infiltration and edema, compared to the control group

Through qualitative analysis in HE, the control group (Fig. 2a and b) exhibited moderate chronic inflammatory infiltrate, represented by the presence of lymphocytes, plasma cells, and macrophages and accompanied by extensive edema. The formation of the angiogenesis process was also observed along with little formations of immature muscle fibers.

The dexamethasone groups (Fig. 2c and d) and copaiba oil-resin (Fig. 2e and f) showed similar aspects to each other, but when compared to the control group showed less chronic inflammatory infiltrate and greater formation of muscle fibers in the injury area. Thus, both treatments showed anti-inflammatory action and accelerated the repair in the area.

The histological sections of the dexamethasone group showed less edema, which did not occur in the control group or in the copaiba oil-resin group. Neither group appeared to have the presence of fibrosis or necrosis.

### Semi-quantitative analysis of inflammatory infiltrate and edema

**Fig. 3:**
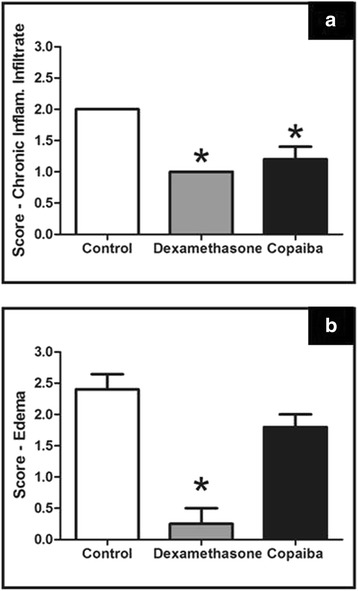
Effect of treatment with copaiba oil-resin on transfixing lesions in rats’ tongues comparing intensities of chronic inflammatory infiltrate and edema. The results are expressed as mean ± SE of the scores attributed to each variable analyzed. Panel (**a**): variable chronic inflammatory infiltrate; Panel (**b**): variable edema (*n* = 5 animals per group). *p ≤ .05 compared to control group (Kruskal-Wallis test, followed by Dunn test)

From the analysis score shown in Fig. 3, treatment with copaiba oil-resin in the proposed model was able to demonstrate anti-inflammatory action, it decreased the intensity of chronic inflammatory infiltrate (1.2 ± 0.20) compared to the control group (2.0 ± 0.0), and its performance was similar to the dexamethasone group (1.0 ± 0.0).

When evaluating the variable edema from the analysis score, we found treatment with the copaiba oil-resin even reduced the intensity of edema (1.8 ± 0.20) but it was not a statistically significant difference compared to the control group (2.4 ± 0:24). Treatment with dexamethasone also decreased the intensity of edema (0:25 ± 0:25) compared to the control group; however, there was no difference from the group treated with copaiba oil-resin.

### Immunohistochemistry analysis

**Fig. 4:**
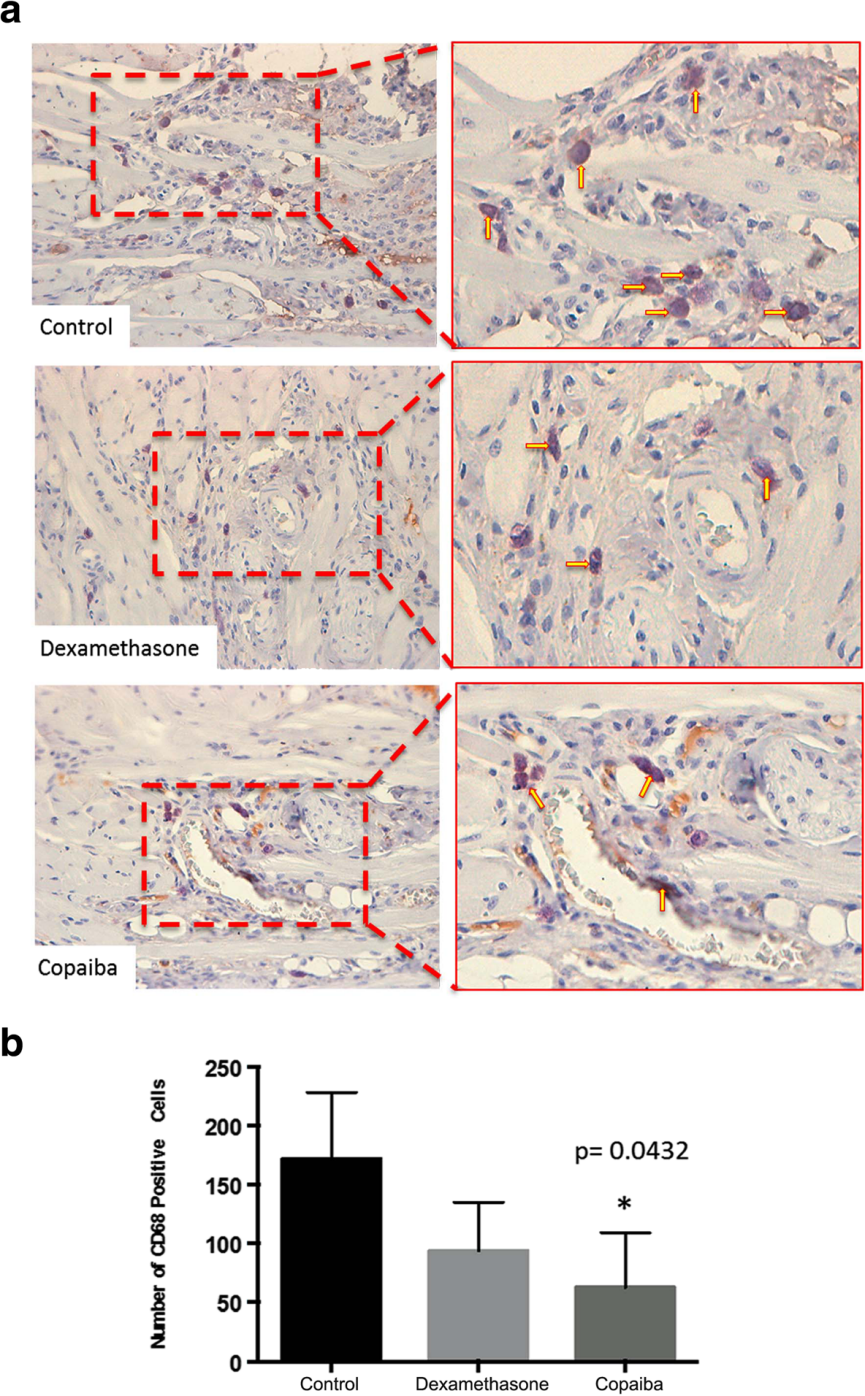
Effect of treatment with copaiba oil-resin on transfixing lesions in rats’ tongues comparing the CD68-positive cells. **a** Photomicrographs showing CD68-positive cells (*arrows*) in control, dexamethasone and oil copaiba Groups. **b** Graphic illustrating the number of CD68-positive cells in three different treatments (*n* = 5 animals per group). *p ≤ .05 compared to control group (one-way ANOVA with Tukey’s post-hoc test)

The treatment with dexamethasone and copaiba oil-resin reduced the number of CD68 positive macrophages. However, a significant reduction was only observed in the copaiba oil-resin group compared to the control group (*p* = .0432, Fig. 4).

## Discussion

This study was the first to investigate the effects of copaiba oil-resin on factors that modulate inflammation of oral tissue. Research has demonstrated that copaiba oil-resin (*Copaifera reticulata Ducke*) could modulate the inflammatory process by decreasing the recruitment of inflammatory cells after 7 days of oral treatment.

Regeneration of integrity and tissue homeostasis after injury is a fundamental property and one of the most complex biological processes that occur during the life of all organisms. Mammals’ response to injury requires a complex and dynamic interaction of numerous cellular and humoral cascades that have the purpose of performing a sequence of repair steps, comprising inflammation, tissue proliferation, and maturation of tissue [22–25].

Inflammation comprises the first phase of tissue repair where neutrophils are recruited immediately after injury and persist in large numbers through the first 48 h after the injury. Then, monocytes arise from the bloodstream and differentiate into macrophages, which phagocytize bacteria and dead cells under the influence of other cellular elements, such as lymphocytes. These inflammatory cells are crucial to the healing process [22, 23]. Thus, the model used in this study mainly assessed the chronic inflammatory infiltrate since the animal’s survival period after injury was 7 days, and was, therefore, composed of lymphocytes, plasma cells, and macrophages.

The modulation of the inflammation is fundamentally important because, during the inflammatory phase, there is a release of growth factors that induce proliferation of fibroblasts with a consequent production of extracellular matrix and myofibroblasts, which are the basis for the healing/reshuffle [22, 24].

Different therapeutic protocols, such as analgesics, corticosteroids, and anti-inflammatory agents have been tested to accelerate the wound healing process. In addition to these drugs, the pharmaceutical industry has focused research on alternative medicine, and it takes as its first ground empirical knowledge of needy populations who use nature’s products as needed to treat their diseases [25–28].

In this scenario, products arising from the Amazon region, many of which have unknown composition, arise as possible alternatives for future drug formulations. Among these, one that is most prominent is copaiba oil-resin. Copaiba oil-resin is a natural substance obtained from the trunk of the *copaifera* tree, originally from the Amazon rainforest, and has been used for over 500 years in traditional folk medicine in the region with great diversity of applications.

This natural substance is obtained by drilling the trunk of the *copaifera* tree, which belongs to the family Leguminosae-Caesalpiniaceas, featuring a variety of species. One that stands out in the Amazon is *Copaifera reticulated Ducke* [2, 29, 30], the same used in this investigation.

The method of testing for acute oral toxicity, as used in our study, is among the most currently accepted in literature [26]. Although other studies have used other methods also advocated by the OECD, they observed that copaiba oil-resin intake by the rat species used in our investigation caused no acute oral toxicity, confirming our findings and, once again, demonstrating safe use of this extract by mouth [24, 25].

The lesion induction method is a model developed in our laboratory which can identify the repair process in all the extension of the organ, since the gripper Ainsworth damage all tissues that constitute language (epithelial tissue, connective and muscle). We can observe in the literature other types of induction of injury in the rat tongue using punch [20] and mucositis induction [21], however, neither described able to reach all tissues that constitute tongue.

Copaiba oil-resin’s modulating effect on inflammation that presented in the transfixing injury in rats’ tongues may be attributed to sesquiterpene β-caryophyllene. This compound represents about 40% of copaíba oil-resin’s composition as reported in the literature [2]. The oil-resin used in our survey has 37% of β-caryophyllene.

The literature has shown that this high β-caryophyllene content is primarily responsible for the anti-inflammatory characteristic of copaíba oil-resin [16], as it has been reported that this compound performs selective binding and inhibiting in canabioide receptor type 2 (CB 2). The activation of CB 2 receptors are involved in stimulating the secretion of pro-inflammatory cytokines and increasing T and B lymphocyte response [31, 32].

Our results demonstrate that copaiba oil-resin reduced the number of inflammatory cells after 7 days of injury, which is similar to that found in animals treated with dexamethasone. Dexamethasone anti-inflammatory activity has been well-described in the literature and corresponds to adhesive inhibition effects and migration of leukocytes, inducing apoptosis of inflammatory cells, reduction of edema, and suppression of the production of inflammatory mediators by inhibiting phospholipase A2 and induction of anti-inflammatory cytokine IL-10 [33]. However, steroids have been associated with numerous local and systemic side effects [34], and thus the use of other agents, such as copaiba oil-resin oil, reduces inflammation and accelerates cellular repair without causing adverse effects.

The difference in the formation of edema found in treatments with dexamethasone and copaiba oil-resin is because of differences between the two anti-inflammatory mechanisms of the treatments. However, regardless of the amount of swelling, both groups had reduced numbers of inflammatory cells and favored the formation of new fibers.

## Conclusion

The results indicate that copaiba oil-resin is a natural product effective in reducing chronic inflammatory infiltrate and inhibiting macrophage activity. For not presenting effectiveness of reduction of edema, our data direct to new investigations that seek to elucidate the role of this oil-resin on other mechanisms associated with inflammation.

## Abbreviations

CB 2: Canabioide receptor type
Cox-2: Cyclooxygenase-2
DAB: Diaminobenzidine tetrahydrochloride
HE: Hematoxylin and eosin
OECD: Organization for Economic Co-operation and Development
UFPA: Federal University of Pará
